# Postoperative outcomes in patients with diabetes after enhanced recovery thoracoscopic lobectomy

**DOI:** 10.1007/s00464-024-10936-2

**Published:** 2024-06-07

**Authors:** Lin Huang, René Horsleben Petersen, Henrik Kehlet

**Affiliations:** 1 Department of Cardiothoracic Surgery, Copenhagen University Hospital, Rigshospitalet, Copenhagen, Denmark; 2 Section for Surgical Pathophysiology, Copenhagen University Hospital, Rigshospitalet, Blegdamsvej 9, 2100 Copenhagen, Denmark

**Keywords:** Enhanced recovery after surgery, Diabetes, Video-assisted thoracoscopic surgery, Lobectomy, Comorbidity, Mortality

## Abstract

**Background:**

Diabetes is considered a general surgical risk factor, but with few data from enhanced recovery (ERAS) otherwise known to improve outcome. Therefore, this study aimed to investigate postoperative outcomes of patients with diabetes who underwent video-assisted thoracoscopic surgery (VATS) lobectomy in an established ERAS setting.

**Methods:**

We retrospectively analysed outcome data (hospital stay (LOS), readmissions, and mortality) from a prospective database with consecutive unselected ERAS VATS lobectomies from 2012 to 2022. Complete follow-up was secured by the registration system in East Denmark.

**Results:**

We included 3164 patients of which 323 had diabetes, including 186 treated with insulin and antidiabetic medicine, 35 with insulin only and 102 with antidiabetic medicine only. The median LOS was 3 days, stable over the study period. There were no differences in terms of LOS, postoperative complications, readmissions or 30 days alive and out of hospital. Patients with diabetes had significantly higher 30- and 90-day mortality rates compared to those without diabetes (*p* < .001), but also had higher preoperative comorbidity. Preoperative HbA1c levels did not correlate with postoperative outcomes.

**Conclusion:**

In an ERAS setting, diabetes may not increase the risk for prolonged LOS, complications, and readmissions after VATS lobectomy, however with higher 30- and 90-day mortality probably related to more preoperative comorbidities.

**Supplementary Information:**

The online version contains supplementary material available at 10.1007/s00464-024-10936-2.

Diabetes continues to pose a significant challenge to public health [1]. When addressing patients with diabetes requiring surgical treatment, the risks associated with infection, respiratory, cardiac, renal, cerebral, and vascular complications have consistently been reported to be higher compared to individuals without diabetes [2]. However, the role of diabetes per se as an independent risk factor adjusted for comorbidities and across all surgical procedures remains uncertain [2, 3] as well as the optimal perioperative diabetes management [4, 5]. This uncertainty is particularly notable within the context of the implementation of Enhanced Recovery After Surgery (ERAS) programmes which minimise the undesirable metabolic effects of surgical injury and subsequently decreasing postoperative complications and shortening the length of hospital stay (LOS) through accelerated recovery [6, 7], and therefore of potential major consequences for patients with diabetes considered at “high risk”.

Regarding pulmonary surgery, diabetes has been associated with the occurrence of bronchopleural fistula [8], and inadequately managed diabetes has been linked to increased morbidity [2]. However, these studies did not implement ERAS interventions and did not focus on the use of video-assisted thoracoscopic surgery (VATS) which otherwise may improve outcome [9, 10]. Moreover, the existing publications have mostly aimed on cancer survival prognosis in patients with diabetes following pulmonary cancer resection [11, 12], rather than procedure-specific data on early postoperative outcomes.

Therefore, the aim of this study was to examine LOS, postoperative complications within 30 days after surgery, 30-day readmissions, as well as 30- and 90-day mortality among pharmacologically managed patients with diabetes who underwent VATS lobectomy within an established ERAS programmes with a median LOS of 3 days within a 10-year period and compared to patients without diabetes.

## Methods

### Study design

The study had a retrospective, observational design utilising data from a prospective institutional database from consecutive VATS lobectomy cases at Rigshospitalet, Copenhagen. Compliance with Danish regulations was approved from both the Review Board in the Central Region of Denmark (IRB, R-23033205) and the Danish Data Protection Agency (P-2023-14323) before the study commencement and the requirement for written informed consent was waived by the IRB. Study report adhered to the Strengthening the Reporting of Observational Studies in Epidemiology statement (STROBE) [13].

### Data source

The Department of Cardiothoracic Surgery at Rigshospitalet is a tertiary hospital in East Denmark that has implemented an ERAS setup for lung surgery for about 10 years [14] and being one of the largest-volume European VATS centres. The ERAS setting has been published before [15] and summarised in Supplementary Table 1. The perioperative diabetes management followed the Danish national guidelines (https://endocrinology.dk/nbv/diabetes-melitus/diabetes-og-kirurgi/), but individual details were not recorded.

Within our database, demographic information, perioperative clinical data, and follow-up details were consistently and prospectively updated by research nurses through the utilisation of the electronic medical record system (E-journal), ensuring a complete enrolment process and comprehensive follow-up for economic reimbursement considerations. Readmission and mortality were assessed from the Danish CPR-register and reasons for both from hospital records. The operational workflow was supported using an electronic healthcare software program (Epic, Madison, WI, USA). The electronic record organisation in East Denmark secured complete follow-up wherever a readmission occurred and where the thoracic department is the only surgery centre. Subsequently, all information was stored in Excel files.

### Patients

All patients who underwent VATS lobectomy between the years 2012 and 2022 were included. Exclusion criteria were patients < 18 years, individuals residing outside East Denmark and those with a history of neoadjuvant oncological therapy. Additionally, patients with diabetes who did not receive pharmacological management with insulin or other antidiabetic medication were excluded.

### Variables

Patient characteristics for analyses included age, gender, percentage of predicted forced expiratory volume in 1s (FEV_1_%_pre_), smoking status, comorbidities, surgical duration, blood loss, and pathology. Preoperative haemoglobin A1c (HbA1c) values were recorded from the electronic records and were taken median 71 (interquartile range [IQR] 42–152) days before surgery.

The primary outcomes were early postoperative outcomes, including LOS (counted as number of overnight stays in Rigshospitalet or other hospitals in East Denmark if patients were transferred), complications within 30 days postoperatively, 30-day readmissions (defined as any admission related to the index procedure with overnight stay). The secondary outcomes were 30 days alive and out of hospital (DAOH-30), 30- and 90-day mortality and reasons of death in relation to the preoperative type of pharmacological diabetes management.

### Statistics

No formal power calculation for sample size in this retrospective, observational study was employed. There was no missing data in any variable. Continuous variables are presented as median and IQR owing to non-normal distribution identified by the Kolmogorov–Smirnov and the Shapiro–Wilk tests, except for LOS and duration of chest drainage presented as median and IQR and mean ± standard deviation (SD). Categorical variables are presented as number and percentage. Differences of primary outcomes between patients with and without diabetes are compared by t-test/Mann–Whitney U test or Chi-squared/Fisher’s exact test where appropriate. A logistic regression model including all patient demographics was used to assess the association of pharmacological diabetes management with LOS, readmissions, and mortality. Two-side *P* < 0.05 was used for statistical difference. All analyses were completed by R Software (version 4.3.1 R Foundation for Computing, Vienna, Austria).

## Results

**Fig. 1:**
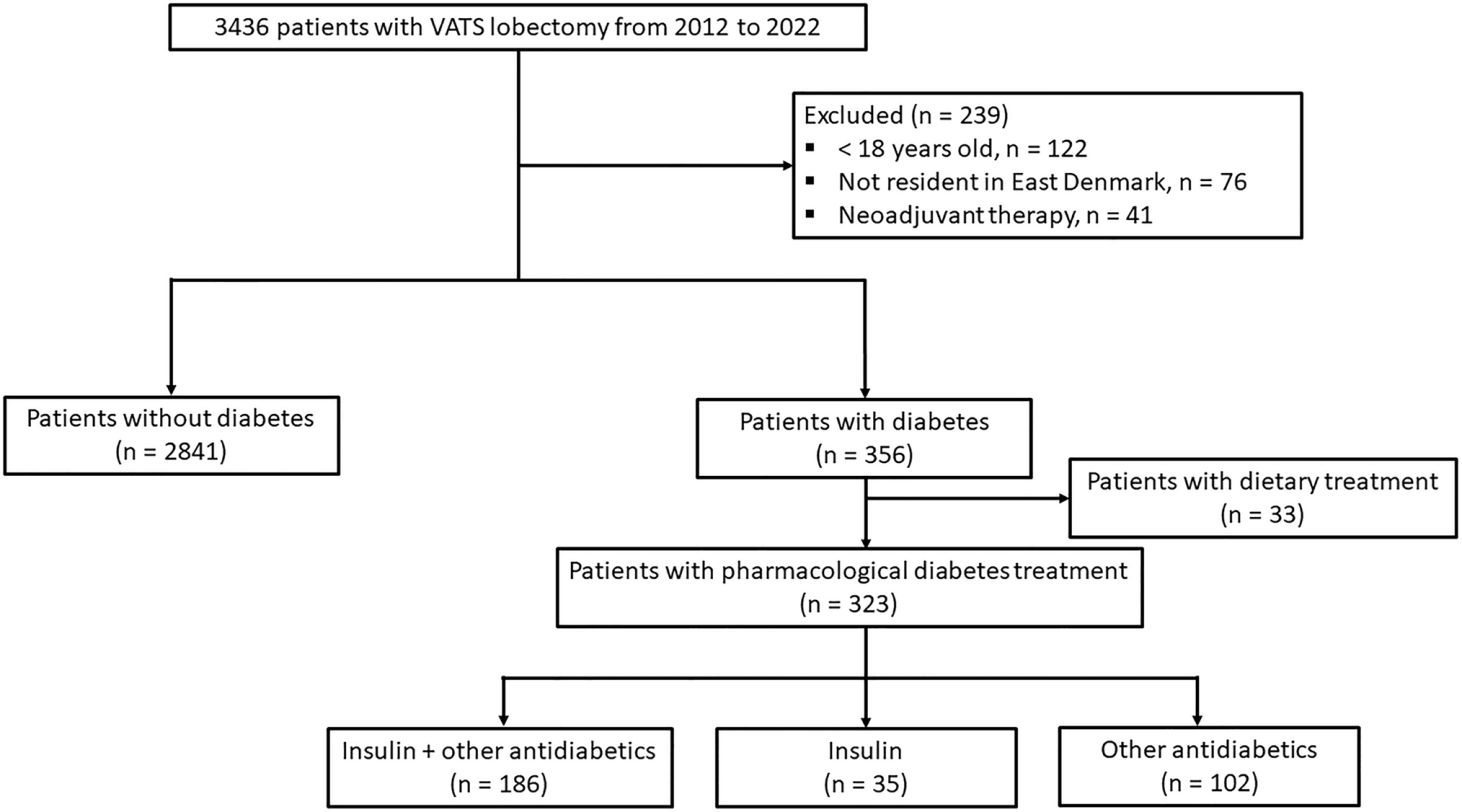
Patient enrolment. *VATS* video-assisted thoracoscopic surgery

Of 3436 patients with ERAS VATS lobectomy, 3164 fulfilled the inclusion criteria for final analyses. There were 323 patients with pharmacological diabetes treatment, including 186 treated by insulin and other antidiabetics, 35 only with insulin and 102 only with other antidiabetics. (Fig. 1).

The diabetes group had more males and older patients, a longer history of smoking, reduced FEV_1_%_pre_, more cardiovascular, renal/urinary, hepato-pancreatic, and hypertension comorbidities when compared to patients without diabetes (Table 1).

**Table 1:** Patient characteristics

Variables	Patients without diabetes (*n* = 2841)	Patients with diabetes (*n* = 323)	*P*-value^a^	Insulin + other antidiabetics (*n* = 186)	*P*-value^b^	Insulin (*n* = 35)	P-value^c^	Other antidiabetics (*n* = 102)	*P*-value^d^
Age, year, median (IQR)	69 (62–75)	71 (65–75)	**0.004**	71 (65–75)	**0.021**	69 (62–76)	0.721	71 (65–75)	0.055
Gender, n (%)			** < 0.001**		** < 0.001**		0.134		**0.008**
Male	1185 (41.7%)	182 (56.3%)		107 (57.5%)		19 (54.3%)		56 (54.9%)	
Female	1656 (58.3%)	141 (43.7%)		79 (42.5%)		16 (45.7%)		46 (45.1%)	
FEV_1_%pre, median (IQR)	87 (73–101)	82 (71–96)	** < 00.001**	82 (70–96)	**0.005**	83 (74–97)	0.437	83 (70–93)	**0.043**
Smoking status, n (%)			**0.001**		**0.004**		0.924		0.151
Never	468 (16.5%)	29 (9.0%)		14 (7.5%)		5 (14.3%)		10 (9.8%)	
Former smoker	1303 (45.9%)	154 (47.7%)		90 (48.4%)		17 (48.6%)		47 (46.1%)	
Current smoker	1070 (37.7%)	140 (43.3%)		82 (44.1%)		13 (37.1%)		45 (44.1%)	
Comorbidities, n (%)									
Pulmonary	703 (24.7%)	92 (28.5%)	0.142	52 (28.0%)	0.327	8 (22.9%)	1.000	32 (31.4%)	0.129
Cardiovascular	634 (22.3%)	126 (39.0%)	** < 0.001**	77 (41.4%)	** < 0.001**	10 (28.6%)	0.0378	39 (38.2%)	** < 0.001**
Renal/Urinary	68 (2.4%)	23 (7.1%)	** < .001**	15 (8.1%)	** < 0.001**	5 (14.3%)	**0.002**	3 (2.9%)	.723
Hepato-pancreatic	60 (2.1%)	16 (5.0%)	**0.002**	10 (5.4%)	**0.004**	2 (5.7%)	0.173	4 (3.9%)	0.282
Cerebral	202 (7.1%)	31 (9.6%)	0.105	15 (8.1%)	.625	2 (5.7%)	1.000	14 (13.7%)	**0.012**
Limb	242 (8.5%)	25 (7.7%)	0.634	14 (7.5%)	0.638	5 (14.3%)	0.219	6 (5.9%)	0.467
Hypertension	985 (34.7%)	209 (64.7%)	** < 0.001**	120 (64.5%)	** < 0.001**	22 (62.9%)	** < .001**	67 (65.7%)	** < 0.001**
History of cancer	1168 (41.1%)	127 (39.3%)	0.535	74 (39.8%)	0.721	12 (34.3%)	0.414	41 (40.2%)	0.853
Surgical duration, min, median (IQR)	95 (80–116)	103 (85–120)	** < 0.001**	104 (85–123)	** < 0.001**	103 (80–115)	0.489	100 (83–125)	0.087
Blood loss, ml, median (IQR)	25 (10–75)	50 (20–100)	** < 0.001**	50 (20–105)	** < 0.001**	50 (20–150)	**0.009**	50 (20–100)	**0.011**
Lobe resected, n (%)			0.730		.309		.687		.714
LUL	633 (22.3%)	66 (20.4%)		37 (19.9%)		5 (14.3%)		24 (23.5%)	
LLL	424 (14.9%)	53 (16.4%)		31 (16.7%)		4 (11.4%)		18 (17.6%)	
RUL	961 (33.9%)	115 (35.6%)		74 (39.8%)		13 (37.1%)		28 (27.5%)	
RML	209 (7.4%)	27 (8.4%)		15 (8.1%)		2 (5.7%)		10 (9.8%)	
RLL	552 (19.4%)	54 (16.7%)		25 (13.4%)		10 (28.6%)		19 (18.6%)	
Bi	60 (2.1%)	8 (2.5%)		4 (2.2%)		1 (2.9%)		3 (2.9%)	
Pathology, n (%)			0.270		0.306		0.615		0.065
Primary lung cancer	2545 (89.6%)	297 (92.0%)		173 (93.0%)		33 (94.3%)		91 (89.2%)	
Metastasis	199 (7.0%)	20 (6.2%)		8 (4.3%)		1 (2.9%)		11 (10.8%)	
Benign	97 (3.4%)	6 (1.9%)		5 (2.7%)		1 (2.9%)		0 (0.0%)	
HbA1c, mmol/mol, median (IQR)	–	51 (46–59)	–	52 (47–60)	–	58 (46–67)	–	48 (44–54)	–

Bold values indicate difference in comparison

*Bi* bilobectomy; FEV_1_%_pre_: percentage of predicted forced expiratory volume in 1s; *HbA1c* haemoglobin A1c; *IQR* interquartile range; *LLL* left lower lobe; *LUL* left upper lobe; *RLL* right lower lobe; *RML* right middle lobe; *RUL* right upper lobe

^a^Non-diabetics vs. diabetics

^b^Non-diabetics vs. diabetics treated by insulin and other antidiabetics

^c^Non-diabetics vs. diabetics treated by insulin

^d^Non-diabetics vs. diabetics treated by other antidiabetics

Median postoperative LOS was 3 days in the 10-year study period regardless of the presence of diabetes or the type of pharmacological diabetes management (Table 2). Mean LOS was also similar among the different groups, as well as median and mean duration of chest drainage and 30-day postoperative complications and 30-day readmissions (Table 2). In contrast, the 30-day mortality rate was higher in patients with diabetes than without (4.0% vs. 0.8%) (*p* < 0.001). Also, the 90-day mortality rate in the group with diabetes was higher compared to the group without diabetes (4.3% vs. 1.7%) (*p* < 0.001), and stable during the study period. When compared with patients without diabetes, the 30-day and 90-day mortality rate was higher in the group with diabetes treated only with insulin (*p* < 0.001, *p* = 0.003) and in the group treated only with other antidiabetics (*p* = 0.002, *p* = 0.032) (Table 2).

**Table 2:** Postoperative outcomes

Variables	Patients without diabetes (*n* = 2841)	Patients with diabetes (*n* = 323)	*P*-value^a^	Insulin + other antidiabetics (*n* = 186)	*P*-value^b^	Insulin (*n* = 35)	P-value^c^	Other antidiabetics (*n* = 102)	*P*-value^d^
LOS, day									
Median (IQR)	3 (2–6)	3 (2–8)	0.633	3 (2–8)	0.482	3 (2–8)	0.268	3 (2–6)	0.468
Mean ± SD	5 ± 7	6 ± 6	0.815	6 ± 6	0.549	6 ± 6	0.655	5 ± 5	0.454
Duration of chest drainage, day									
Median (IQR)	2 (1–4)	2 (1–4)	0.605	2 (1–4)	0.604	1 (1–4)	0.699	2 (1–3)	0.903
Mean ± SD	4 ± 5	3 ± 4	0.115	3 ± 4	0.309	4 ± 4	0.787	3 ± 4	0.138
Patients with 30-day complications, n (%)	1001 (35.2%)	120 (37.2%)	0.495	71 (38.2%)	0.417	14 (40.0%)	0.558	35 (34.3%)	0.848
Patients with 30-day readmissions, n (%)	391 (13.8%)	47 (14.6%)	0.697	29 (15.6%)	0.485	4 (11.4%)	1.000	14 (13.7%)	0.991
DAOH30, day, median (IQR)	27 (22–28)	26 (20–28)	0.330	26 (20–28)	0.282	25 (19–28)	0.111	27 (21–28)	0.966
30-day mortality, n (%)	23 (0.8%)	13 (4.0%)	** < 0.001**	4 (2.2%)	0.080	4 (11.4%)	** < 0.001**	5 (4.9%)	**0.002**
90-day mortality, n (%)	47 (1.7%)	14 (4.3%)	** < 0.001**	5 (2.7%)	0.248	4 (11.4%)	**0.003**	5 (4.9%)	**0.032**

Bold values indicate difference in comparison

*DAOH30* days alive and out of hospital within postoperative 30 days; *IQR* interquartile range; *LOS* length of stay; *SD* standard deviation

^a^Non-diabetics vs. diabetics

^b^Non-diabetics vs. diabetics treated by insulin and other antidiabetics

^c^Non-diabetics vs. diabetics treated by insulin

^d^Non-diabetics vs. diabetics treated by other antidiabetics

Despite the mortality difference the all over DAOH-30 was not significantly different between groups (Table 2).

Regarding specific types of complications (Table 3), no significant difference was found between the two groups.

**Table 3:** Specific 30-day complications after video-assisted thoracoscopic surgery lobectomy

Variables, number of patients with complications (% of all). One patient may have one or more complications	Patients without diabetics *n* = 1001/2841 (35.2%)	Patients with diabetics *n* = 120/323 (37.2%)	Insulin + other antidiabetics *n* = 71/186 (38.2%)	Insulin *n* = 14/35 (40.0%)	Other antidiabetics *n* = 35/102 (34.3%)
Infection					
Pneumonia	452 (15.9)	60 (18.6)	39 (21.0)	6 (17.1)	15 (14.7)
Empyema	73 (2.6)	7 (2.2)	4 (2.2)	1 (2.9)	2 (2.0)
Urinary tract infection	37 (1.3)	5 (1.5)	4 (2.2)	1 (2.9)	0 (0.0)
Sepsis	18 (0.6)	7 (2.2)	3 (1.6)	1 (2.9)	3 (2.9)
Wound infection	58 (2.0)	7 (2.2%)	5 (2.7)	1 (2.9)	1 (1.0)
Pulmonary and thoracic complications					
Pneumothorax	247 (8.7)	20 (6.2)	12 (6.5)	2 (5.7)	6 (5.9)
Pleural effusion	80 (2.8)	14 (4.3)	9 (4.8)	0 (0.0)	5 (4.9)
Respiratory insufficiency	40 (1.4)	12 (3.7)	9 (4.8)	1 (2.9)	2 (2.0)
Bronchopleural fistula	3 (0.1)	0 (0.0)	0 (0.0)	0 (0.0)	0 (0.0)
Others*	27 (1.0)	5 (1.5)	3 (1.6)	1 (2.9)	1 (1.0)
Cardiac complications					
Atrial fibrillation	225 (7.9)	26 (8.0)	18 (9.7)	1 (2.9)	7 (6.9)
Myocardial infarction/cardiac failure	8 (0.3)	3 (0.9)	2 (1.1)	1 (2.9)	0 (0.0)
Other complications					
Postoperative bleeding	58 (2.0)	10 (3.1)	3 (1.6)	3 (8.6)	4 (3.9)
Gastrointestinal bleeding/ulcer	23 (0.8)	2 (0.6)	0 (0.0)	1 (2.9)	1 (1.0)
Ileus	11 (0.4)	4 (1.2)	2 (1.1)	1 (2.9)	1 (1.0)
Stroke	15 (0.5)	7 (2.2)	4 (2.2)	1 (2.9)	2 (2.0)
Confusion and delirium	20 (0.7)	5 (1.5)	4 (2.2)	0 (0.0)	1 (1.0)
Kidney insufficiency	23 (0.8)	7 (2.2)	4 (2.2)	0 (0.0)	3 (2.9)
Urinary retention	10 (0.4)	2 (0.6)	2 (1.1)	0 (0.0)	0 (0.0)

*Others in pulmonary and thoracic complications included chylothorax, haemopneumothorax, and pulmonary embolism

Pneumonia (5.0%), pleural effusion (1.9%), pneumothorax (1.5%), and urinary tract infection (1.2%) were the dominant factors leading to 30-day readmission in the diabetic group compared with pneumothorax (4.5%), pneumonia (4.4%), atrial fibrillation (1.1%) in the non-diabetic group (Supplementary Table 2).

For patients without diabetes, the main reasons for death within 30 days postoperatively were severe pneumonia (0.4%) and respiratory failure (0.2%), followed by bronchopleural fistula (0.1%) or unexplained (0.1%). In patients with diabetes, the most dominant reasons for 30-day mortality were severe pneumonia (1.2%), intestinal ischaemia (0.6%), one patient (0.3%) due to pancreatic cancer or unexplained (0.3%) (Supplementary Table 3). Regarding deaths that occurred between 31 and 90 days after surgery, only one patient with diabetes died due to kidney failure, while there were various reasons for the patients without diabetes who died during this period (Supplementary Table 3). There was no difference in pre- and intraoperative demographics between diabetes patients who survived or not, but the limited number of deaths precluded a more detailed analysis. However, the patients with diabetes who died had more 30 days complications (92.9%) vs. 34.6% who did not die (*p* < 0.001) (Supplementary Table 4).

There was no difference in the proportion of preoperative HbA1c values (> 64 mmol/mol) in those who had complications or not (42.3% vs. 57.7%, *p* = 0.401) or between surviving or not surviving (15.9% vs. 21.4%, *p* = 0.479).

The adjusted logistic regression model including preoperative demographics showed that pharmacological treated diabetes did not have a significant impact on LOS, postoperative 30-day complications, or readmissions. However, insulin treatment per se was associated with increased risks in both 30-day mortality (odds ratio (OR) 13.2, 95% confidence interval (95% CI) 3.8 to 45.8, *p* < 0.001) and 90-day mortality (OR 5.1, 95% CI 1.7 to 15.0, *p* = 0.003). Similarly, other antidiabetic medicine treatment was associated with a higher risk of 30-day mortality (OR 6.9, 95% CI 2.2 to 22.1, *p* < 0.001).

## Discussion

This first available retrospective observational study found that in patients who underwent VATS lobectomy within an effective ERAS programme, pharmacologically treated diabetes did not result in prolonged LOS, higher rates of 30-day postoperative complications, or increased incidence of 30-day readmissions. However, it was associated with higher 30- and 90-day mortality rates compared to patients without diabetes.

To date, only two studies have investigated early postoperative outcomes in patients with diabetes within an implemented ERAS programme [16, 17]. In colorectal surgery, no difference was reported between groups with and without diabetes in terms of postoperative complications and mortality [17]. However, in fast-track hip and knee arthroplasty, pharmacologically treated diabetes was associated with a slightly higher risk of prolonged LOS, but without increased mortality [16]. Nevertheless, in insulin treated patients more diabetes-related morbidities occurred after fast-track hip and knee arthroplasty, particularly concerning cardiac complications. This discrepancy may result from differences in the surgical population demographics or the perioperative diabetes management, the latter being debatable and with limited high-quality evidence for recommendations [4].

One challenge when interpreting the diabetic surgical literature is the role of the preoperative diabetes management which often is not presented. In the current study, we divided between different types of diabetic treatment (insulin vs. other antidiabetics or combinations), but where the relatively small subgroups didn’t show any differences in outcome, neither regarding preoperative HbA1c status.

In the present study with 10-year database registration with a stable fully implemented ERAS programme with a median LOS of 3 days, the conclusion that diabetes did not appear to be a specific risk factor for LOS, complications, and readmission is in accordance with our recent studies from the last couple of years, and not showing any perioperative risk signals for diabetes or reduced days alive and out of hospital after ERAS VATS lobectomy [18, 19]. Also, our results are supported by a large, but less detailed and non-ERAS database study that did not find diabetes to be a risk factor for readmission after lobectomy [20].

The higher postoperative mortality observed in patients with diabetes compared to those without diabetes may be expected, given that patients with diabetes in this study had a higher burden of comorbidities, especially within the pulmonary, cardiovascular, and renal/urinary systems. This observation is supported by a study of HbA1c in non-diabetic patients from unspecific surgeries, where postoperative outcomes were more dependent on end-organ preoperative comorbidity than HbA1c levels [21]. These comorbidity findings are consistent with previous research conducted within an ERAS context for non-lung surgeries [16, 17] or in a non-ERAS context for lung surgeries [11]. Furthermore, our conclusion is supported by a recent large-sample Danish breast cancer study that elucidated the relationship between diabetes and comorbidities on (predominantly wound-related) surgical outcomes [22]. Therefore, future strategies aiming to improve survival for patients with diabetes should focus on the specific perioperative organ support requirements in these high-risk individuals.

In terms of postoperative complications and in contrast to the non-ERAS literature [2], we did not find significant differences between rate and major types of complications between patients with or without diabetes, which potentially may be due to the fully implemented ERAS programme.

Our study may be important being the first procedure-specific study in VATS lobectomy within an ERAS programme and including preoperative HbA1c status, type of preoperative diabetes management and detailed information about complications and reasons for mortality. In our study of VATS lobectomy, we consistently found a median LOS of about 3 days over the 10-year period documenting an effective ERAS programme also compared with 2 recent international RCTs (median LOS 4 days) [9, 10]. Also, the anaesthesiological and surgical ERAS care principles (Supplementary Table 1) have been practically the same over the 10-year period. Thus, we did not implement distinct post-surgical protocols for diabetic and nondiabetic patients concerning their therapy. This study investigated potential outcome challenges faced by diabetic patients and showing that diabetic patients experienced longer surgical durations and greater blood loss. However, the procedure of lobectomy per se may not account for mortality differences between the two groups.

However, the study has several limitations. Firstly, there is a lack of details on perioperative data on diabetes management, which was suggested to follow local guidelines. Notably, our ERAS protocol does not include perioperative carbohydrate treatment, as the benefits of this intervention are still controversial and only supported by low level evidence [4, 23]. Secondly, preoperative HbA1c values were obtained from a variable period preoperatively limiting interpretation and where a closer preoperative value was preferred. Thirdly, we had no data on perioperative glucose levels. However, we should mention that in accordance with local guidelines, all diabetic patients who underwent lobectomy had their blood glucose levels controlled perioperatively. Fourthly, postoperative complications were not evaluated according to the Clavien-Dindo classification, but rather by the prospectively collected database information by nurses. Fifthly, a detailed mortality analysis was not possible since several patients died at home and with an overall limited performance of post-mortem autopsy in Denmark. Sixthly, it is a retrospective study, but with prospective data collection. We did not employ a time-to-event analysis to investigate the impact of diabetes duration on perioperative outcomes due to constraints in our dataset. Finally, it included a rather limited number of patients with diabetes especially in the subgroups not allowing more detailed statistical analyses with propensity score matching. However, the strength of our study is a stable ERAS programme over 10 years with documented short LOS and secondly the Danish National Register System to secure a complete follow-up and furthermore supported by the high-volume surgical unit being the only department of thoracic surgery in East Denmark.

## Conclusion

Within a fully implemented ERAS programme for VATS lobectomy, diabetes was not found to increase risk for prolonged LOS, 30-day postoperative complications or readmission, however with a higher 30- and 90-day mortality compared to patients without diabetes, probably related to more preoperative comorbidities in the patients with diabetes.

### Supplementary Information

Below is the link to the electronic supplementary material.

Supplementary file1 (DOCX 25 KB)
