# Supplementary material for: Postoperative outcomes in patients with diabetes after enhanced recovery thoracoscopic lobectomy
Source: Surg Endosc. 2024 Jun 7;38(8):4207–14. doi: 10.1007/s00464-024-10936-2 (PMC11289224; doi:10.1007/s00464-024-10936-2)
Supplement: Supplementary file 1 — Supplementary file1 (DOCX 25 KB) [file 464_2024_10936_MOESM1_ESM.docx]

| **Supplementary table 1. The program of enhanced recovery after lung surgery at Rigshospitalet.** |
| --- |
| **Preoperative period** |
| Preadmission information, education, and counselling |
| Preoperative nutrition and pulmonary function assessment |
| Smoking cessation |
| Preoperative fasting and fluid management |
| Preanesthetic sedation |
| **Intraoperative period** |
| Antibiotic prophylaxis and skin preparation |
| Preventing intraoperative hypothermia |
| Intravenous anesthetics with one-lung ventilation |
| Intraoperative fluid management |
| Video-assisted thoracoscopic surgery with a standardized three-port anterior approach |
| Intercostal nerve block and intercostal catheter |
| A single chest drain CH 24 placed |
| Digital chest drainage system |
| **Postoperative period** |
| Postoperative fluid management |
| Early mobilization management   1. Stand up besides the bed after postoperative 3 hours 2. Walk around the bed and to toilet 3-6 hours postoperatively |
| Opioid-sparing pain management |
| Chest drainage system with a standard suction -2 cmH_2_O |
| Early chest drain removal   1. < 20 ml/h for 12 hours 2. Without upper limitation for serous fluid unless chyle or blood |
| Early urinary catheter removal (up to the first morning after surgery) |
| Pulmonary function physiotherapy |

| **Supplementary table 2. Reasons for readmissions after video-assisted thoracoscopic surgery lobectomy.** | | | | | |
| --- | --- | --- | --- | --- | --- |
| **Variables, number of patients with readmissions (% of all)** | **Non-diabetics**  **n = 391/2841 (13.8 %)** | **Diabetics**  **n = 47/323**  **(14.6 %)** | **Insulin + other antidiabetics**  **n = 29/186**  **(15.6 %)** | **Insulin**  **n = 4/35**  **(11.4 %)** | **Other antidiabetics n = 14/102**  **(13.7 %)** |
| **Reasons for 30-day readmissions** * |  |  |  |  |  |
| Pneumonia | 124 (4.4%) | 16 (5.0%) | 12 (6.5%) | 2 (5.7%) | 2 (2.0%) |
| Pneumothorax | 129 (4.5%) | 5 (1.5%) | 2 (1.1%) | - | 3 (2.9%) |
| Haemothorax/Chylothorax/Pulmonary embolism | 7 (0.2%) | 2 (0.6%) | 1 (0.5%) | - | 1 (1.0%) |
| Empyema | 16 (0.6%) | 2 (0.6%) | 2 (1.1%) | - | - |
| Respiratory insufficiency | 2 (0.1%) | 1 (0.3%) | 1 (0.5%) | - | - |
| Pleural effusion | 28 (1.0%) | 6 (1.9%) | 4 (2.2%) | - | 2 (2.0%) |
| Urinary tract infection | 9 (0.3%) | 4 (1.2%) | 3 (1.6%) | - | 1 (1.0%) |
| Kidney insufficiency | 4 (0.1%) | 1 (0.3%) | 1 (0.5%) | - | - |
| Wound infection | 18 (0.6%) | 2 (0.6%) | 2 (1.1%) | - | - |
| Stroke | 7 (0.2%) | 2 (0.6%) | 1 (0.5%) | - | 1 (1.0%) |
| Atrial fibrillation | 31 (1.1%) | 2 (0.6%) | 1 (0.5%) | - | 1 (1.0%) |
| Myocardial infarction/Cardiac failure | 8 (0.3%) | - | - | - | - |
| Pain | 29 (1.0%) | 3 (0.9%) | - | 1 (2.9%) | 2 (2.0%) |
| Urinary retention/Diarrhea/Constipation | 18 (0.6%) | 2 (0.6%) | 1 (0.5%) | 1 (2.9%) | - |
| Ileus | 1 (0.03%) | 2 (0.6%) | 1 (0.5%) | - | 1 (1.0%) |
| Gastrointestinal bleeding/ulcer | 10 (0.4%) | - | - | - | - |
| Sepsis | 2 (0.1%) | - | - | - | - |
| * one patient can have more than one complication. | | | | | |

| **Supplementary table 3. Main reasons for death after video-assisted thoracoscopic surgery lobectomy.** | | | | | |
| --- | --- | --- | --- | --- | --- |
| **Variables, number of patients with death (% of all)** | **Non-diabetics**  **n = 47/2841**  **(1.7 %)** | **Diabetics**  **n = 14/323**  **(4.3 %)** | **Insulin + other antidiabetics**  **n = 5/186**  **(2.7 %)** | **Insulin**  **n = 4/35**  **(11.4 %)** | **Other antidiabetics n = 5/102**  **(4.9 %)** |
| **0-30 days after surgery** | 23 (0.8%) | 13 (4.0%) | 4 (2.2%) | 4 (11.4%) | 5 (4.9%) |
| Severe pneumonia | 10 (0.4%) | 4 (1.2%) | 2 (1.1%) | 1 (2.4%) | - |
| Pulmonary embolism | 1 (0.04%) | - | - | - | - |
| Bronchopleural fistula | 3 (0.1%) | - | - | - | - |
| Respiratory failure | 5 (0.2%) | 1 (0.3%) | - | - | - |
| Ileus (Ogilvie) | 1 (0.04%) | 1 (0.3%) | - | - | 1 (1.0%) |
| Intestinal ischaemia | - | 2 (0.6%) | - | 1 (2.4%) | - |
| Stroke | - | 2 (0.6%) | - | 1 (2.4%) | - |
| Extrapulmonary cancer | - | 1 (0.3%) | - | 1 (2.4%) | - |
| Unexplained death at home | 3 (0.1%) | 2 (0.6%) | 1 (0.6%) |  | 1 (1.0%) |
| **31-90 days after surgery** | 24 (0.8%) | 1 (0.3%) | 1 (0.6%) | 0 (0.0%) | 0 (0.0%) |
| Severe pneumonia | 5 (0.2%) | - | - | - | - |
| Empyema | 2 (0.1%) | - | - | - | - |
| Bronchopleural fistula | 1 (0.04%) | - | - | - | - |
| Pulmonary embolism | 1 (0.04%) | - | - | - | - |
| Respiratory failure | 3 (0.1%) | - | - | - | - |
| Kidney failure | 1 (0.04%) | 1 (0.3%) | 1 (0.6%) | - | - |
| Side effects of adjuvant therapy/  Recurrence/Metastasis | 6 (0.2%) | - | - | - | - |
| Extrapulmonary cancer | 1 (0.04%) | - | - | - | - |
| Suicide | 1 (0.04%) | - | - | - | - |
| Pancreatitis | 1 (0.04%) | - | - | - | - |
| Ventricular fibrillation | 1 (0.04%) | - | - | - | - |
| Unexplained death at home | 1 (0.04%) | - | - | - | - |

| **Supplementary table 4. Demographics in diabetic patients with and without mortality (0-90 days).** | | | |
| --- | --- | --- | --- |
| **Variables** | **No death**  **(n = 309)** | **Death**  **(n = 14)** | **P value** |
| Age, year, median (IQR) | 70 (65-75) | 72 (66-77) | .373 |
| Gender, n (%) |  |  | 1.000 |
| Male | 174 (56.3%) | 8 (57.1%) |  |
| Female | 135 (43.7%) | 6 (42.9%) |  |
| FEV_1_%pre, median (IQR) | 82 (71-95) | 86 (75-98) | .551 |
| Smoking status, n (%) |  |  | .965 |
| Never | 28 (9.1%) | 1 (7.1%) |  |
| Former smoker | 134 (43.3%) | 6 (42.9%) |  |
| Current smoker | 147 (47.6%) | 7 (50.0%) |  |
| Comorbidities, n (%) |  |  |  |
| Pulmonary | 87 (28.2%) | 5 (35.7%) | .551 |
| Cardiovascular | 118 (38.2%) | 8 (57.1%) | .171 |
| Renal/Urinary | 22 (7.1%) | 1 (7.1%) | 1.000 |
| Hepato-pancreatic | 15 (4.9%) | 1 (7.1%) | .516 |
| Cerebral | 30 (9.7%) | 1 (7.1%) | 1.000 |
| Limb | 25 (8.1%) | 0 (0.0%) | .612 |
| Hypertension | 201 (65.0%) | 8 (57.1%) | .574 |
| History of cancer | 124 (40.1%) | 3 (21.4%) | .262 |
| Surgical duration, min, median (IQR) | 103 (85-122) | 95 (79-111) | .362 |
| Blood loss, ml, median (IQR) | 50 (20-100) | 50 (18-113) | .962 |
| Patients with 30-day complications, n (%) | 107 (34.6%) | 13 (92.9%) | **< .001** |
| Patients with HbA1c > 64 mmol/mol, n (%) | 49 (15.9%) | 3 (21.4%) | .479 |
| Treatment of diabetes, n (%) |  |  | .063 |
| Insulin + other antidiabetics | 181 (58.6%) | 5 (35.7%) |  |
| Insulin | 31 (10.0%) | 4 (28.6%) |  |
| Other antidiabetics | 97 (31.4%) | 5 (35.7%) |  |
| FEV_1_%_pre_: percentage of predicted forced expiratory volume in 1 s; HbA1c: haemoglobin A1c; IQR: interquartile range. | | | |
